# Online reviews, customer Q&As, and product sales: A PVAR approach

**DOI:** 10.1371/journal.pone.0290674

**Published:** 2023-11-17

**Authors:** Miao Feng, Yituo Feng, Yang Li

**Affiliations:** 1 Business School, Shandong Management University, Jinan, China; 2 Management Information Systems, Chungbuk National University, Cheongju, South Korea; 3 Business School, Shandong Normal University, Jinan, China; Jiangsu University, CHINA

## Abstract

Online reviews and customer Q&As have emerged as two vital forms of electronic word-of-mouth (eWOM) that significantly influence consumer decisions in e-commerce. Yet, a comprehensive understanding of the individual and combined roles of these eWOM types in shaping market dynamics remains elusive. This study addresses this research gap by tracking and analyzing three months of eWOM and sales data for 120 laptops on Amazon, comprising 7,205 online reviews, 6,365 customer Q&A questions, and 7,419 answers. Leveraging the Panel Vector Autoregression (PVAR) model and STATA16.0 software, we unravel the intricate dynamics between online reviews, customer Q&As, and laptop sales. The empirical results reveal distinctive influence mechanisms of online reviews and customer Q&As on product sales, with review volume and answer valence positively affecting sales. Importantly, answer volume was found to stimulate online reviews and enhance their valence. Our study elucidates the interplay among online reviews, customer Q&As, and product sales, underscoring the need for future research on multi-type eWOM. Further, the insights gleaned offer valuable guidance for online platforms and retailers to strategize their eWOM management.

## Introduction

In recent decades, the proliferation of electronic word-of-mouth (eWOM) has substantially influenced the digital business environment [1]. As a representative type of eWOM, online reviews have become a significant source of information for consumer purchase decisions [2]. Numerous studies have explored the impact of online reviews on individual behavior and market performance [3, 4]. As online interaction becomes more essential in the digital age, consumers’ demands for eWOM services are no longer restricted to one-way interactions, many platforms have integrated social functions, introducing a two-way interactive function, such as customer Q&As [5].

Amazon first introduced the Q&A feature called “Amazon Answer” in 2012. This Q&A system permits users to ask and answer questions related to the product. More than one user can answer a question, and retailers and consumers can answer the questions. Q&As provide consumers with a new way to learn about a product online. A survey from PowerReviews shows that customer Q&As can dramatically improve retailers’ page traffic and product conversion rates. The customer Q&A function has been widely recognized and used by consumers. According to Taobao, 30 million consumers rely on Q&A sharing to make daily consumption decisions. More than 60% of the questions are answered within 10 minutes through accurate big data matching.

**Fig 1:**
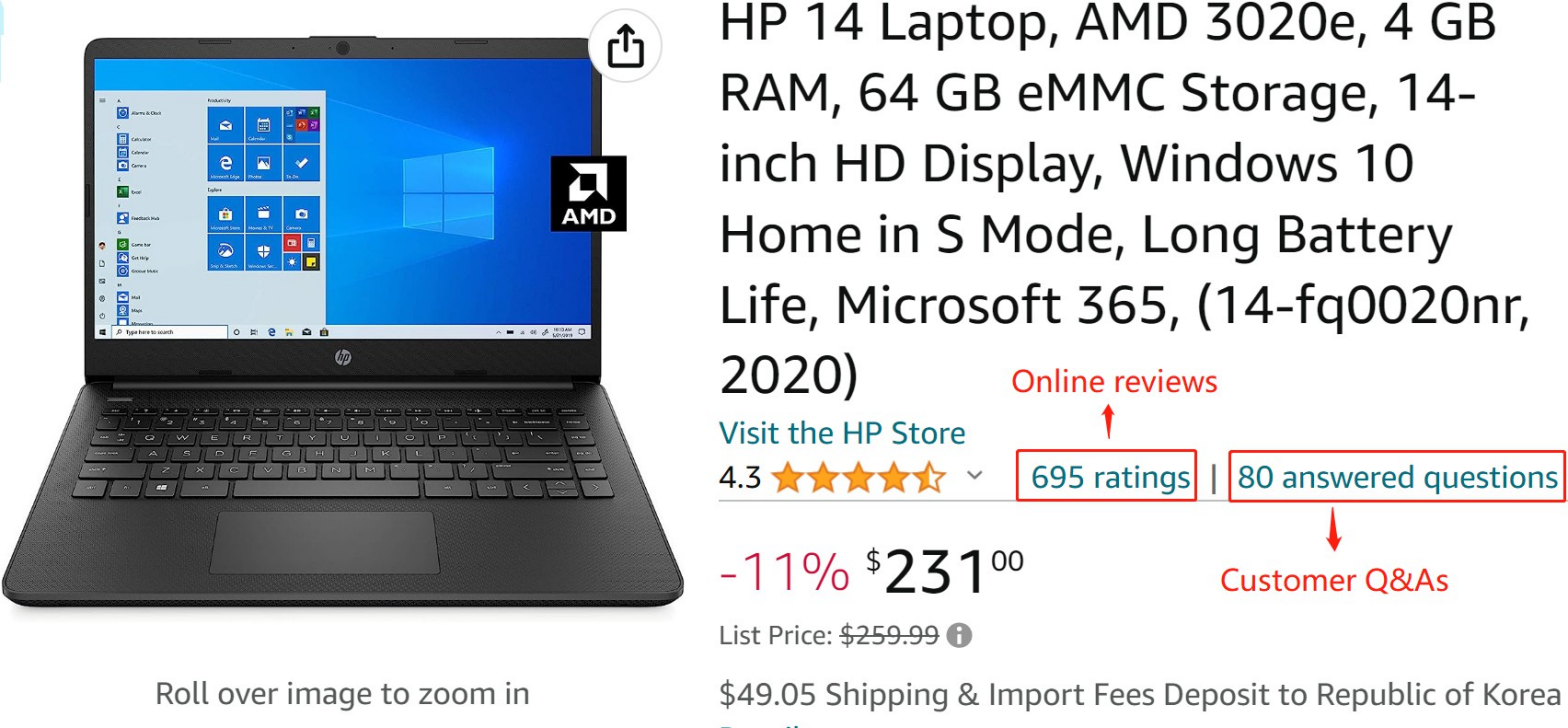
Online reviews and customer Q&As on Amazon.

Online reviews and customer Q&As are the most mainstream type of eWOM. Many e-commerce sites prominently display both types of eWOM (Fig 1 shows an example of online reviews and customer Q&As on Amazon). However, as two representative eWOMs, online reviews and customer Q&As have identified three key differences. First, as mentioned above, online reviews typically provide one-way communication, whereas Q&As are built mainly on two-way interactions. Second, online reviews contain more personal experiences and tend to be more perceptual and abstract. Customer Q&As tend to be related to one (or more) specific function or feature of the product; thus, they are more rational and detailed [6]. Third, online reviews and customer Q&As may play different roles in consumer decision-making. Banerjee et al. [7] proposed that online reviews can decrease product quality uncertainty, whereas customer Q&As can decrease product fit uncertainty.

Based on media richness theory, online reviews and customer Q&A can be viewed as two distinct information mediums, each possessing different degrees of richness. These mediums may play diverse roles in consumer decision-making. Although customer Q&As complement online reviews and enrich the information available to consumers, they may simultaneously cannibalize the online review system, leading to information load [8]. Previous research focused solely on the impact of online reviews and customer Q&As independently and overlooked their joint influence and mutual interdependencies. The economic implications of online reviews and customer Q&As still remain ambiguous. Especially for e-commerce platforms in which information is highly transparent and increasingly integrated with multiple eWOM cues, the intertwined and mutually influential online review and customer Q&A effects are worth studying [9]. To fill the aforementioned research gaps, we present the following research questions:

### Research questions: How do online reviews and customer Q&As impact product sales? How do these two types of eWOM influence each other?

The primary aim of this study is to compare the effects of online reviews and customer Q&As on product sales and to reveal the different mechanisms by which the two types of eWOMs affect consumers. In addition, this study will also explore the interaction between online reviews and customer Q&As. To achieve the research objectives, we collected research data on Amazon, including online reviews, customer Q&As, and a list of the 120 best-selling laptops. To address potential endogeneity issues in research and provide a dynamic analysis, we selected the PVAR model to investigate the interaction among online reviews, customer Q&As, and product sales. The results indicate that review volume and answer valence can positively affect product sales. In addition, answer volume has a positive impact on review volume and review valence. Using impulse response analysis, we further explored the trends in these effects over time.

The study offers two significant contributions. First, to the best of our knowledge, this is the first comprehensive study of online reviews and customer Q&As. The results contribute to the emerging e-commerce Q&A literature and highlight the need for multi-type eWOM research. Second, we explored the dynamic effect of online reviews and Q&As on sales and revealed the interaction relationship between these two types of eWOM. Our empirical results provide academia and practice with a better and more comprehensive understanding of the role of eWOM in consumer decision-making.

The rest of the paper is organized as follows: In Section 2, we provide an overview of previous literature in relevant areas. In Section 3, we describe the data and the variables, and in Section 4, we explain we apply PVAR for the analysis. In Section 5, we report the results. Last, in Section 6, we discuss the results and propose implications for research and practice.

## Literature review

### Informational cascades on E-commerce platforms

When making decisions under uncertainty, individuals will be influenced by others’ decisions and private information. When others’ decisions tend to be consistent, they will give up the contradictory private information and choose to follow others’ decisions, which gives rise to the information cascade phenomenon [10]. Information cascades theory is considered a driver of group behavior when making individual or organizational decisions [11]. For example, when the popularity of a software program reaches a certain level, a large number of online users choose to install the software almost exactly following the behavior of others, with little attention to the objective characteristics of the software [12]. In social networking sites, photos, links, or other information shared by users can easily cause a cascade of re-shares by other friends, eventually making the online content go viral [13].

Informational cascades can also be considered a central driving mechanism to explain the behavior of consumers on e-commerce platforms for the following reasons. First, consumers on e-commerce platforms face decisions under uncertainty because they cannot observe the actual product before purchasing it [2]. Second, most e-commerce platforms provide eWOM systems so that it becomes very convenient for potential consumers to observe eWOM messages from other customers [14]. These eWOM messages in e-commerce platforms largely contain opinions about products and discrete buying recommendations which provide important decision support information for potential consumers [15].

### Media richness theory

Originating from information processing theory, media richness theory suggests that the communication efficiency between individuals is affected by the media matching degree [16]. This theory was primarily used to describe and evaluate the communication medium within an organization, but as communication and the medium involve the transfer and exchange of information and the information behaviors of users, it is also widely used in the field of management information systems and marketing [9]. Media richness is a multidimensional concept that encompasses the number of information cues, interactivity, timeliness, etc [17]. Early studies concluded that the higher the media richness, the more persuasive the message; for example, televisions in video format were considered more persuasive than newspapers in text-only format [18]. However, subsequent studies have found that there are also costs associated with media richness and that combining some modalities may be more detrimental to cognitive processing [19].

When confronted with information from different media, consumers may use multiple cognitive channels to process the information simultaneously, creating an integrated solution [20]. With the development of social media, consumers are exposed to an increasingly diverse range of information in the online environment. Consumer interaction and behavior on word-of-mouth varies significantly across media [21]. Xu et al. [22] found that high media-rich online reviews increase users’ perceptions of usefulness, credibility, and persuasiveness, reinforcing users’ purchase intentions. Besides purchase intentions, media richness was also found to influence the formation of prosocial relationships [23], and satisfaction of online users [24]. In this study, media richness theory can be used to explain the different roles of online reviews and customer Q&A, in consumer decision-making.

### Electronic word-of-mouth and sales

Electronic Word-of-Mouth (eWOM) refers to positive or negative statements made by potential, actual, and former customers about a product or company via the Internet [25]. Evaluating the impact of eWOM on sales has attracted considerable scholarly attention [26, 27]. Previous studies on the effects of eWOM can be classified into two levels: market level and individual level. At the individual level, scholars focus on the impact of information content and the sender’s personal traits on receivers [28]. The primary outcome variables examined include information usefulness [29], eWOM credibility [15], consumer attitude [30], and purchase intention [26]. At the market level, this line of investigation explores the dynamic impact of eWOM messages on product sales using secondary data extracted from websites or review platforms [27].

Many scholars have studied the effect of the volume and valence of online reviews on product sales, however, there is still some disagreement on the conclusions [1]. Volume indicates the total quantity of online interactions [31], while valence represents the consumer’s emotional disposition towards the purchased product, which can be positive or negative [32]. In the study of eWOM, one stream of scholars suggests that the volume of online reviews positively affects product sales because it can reflect the popularity of the product and attract the attention of potential consumers [6, 32]. In contrast, another stream of researchers argues that the main predictor of product sales is not the volume of eWOM, but its valence [26, 33]. As eWOM valence reflects the product’s reputation and quality, it can shape, enhance, or modify potential consumers’ preferences for the product [34].

Although eWOM volume and valence can model the impact of product reviews on sales, the rich information embedded in eWOM cannot be captured in scaler terms [35, 36]. Recently, IS and marketing scholars have paid increasing attention to unstructured eWOM data, that is, the text content of eWOM [37]. Cao et al. [38] emphasized the importance of applying text-mining technology in eWOM research; they empirically demonstrated that semantic characteristics are more influential than other factors in affecting how many reviews receive helpfulness votes. Traditionally, text analysis has been used to analyze the content of eWOM and predict individuals’ psychological states and behaviors [39]. Linguistic Inquiry and Word Count (LIWC) is the most widely employed text-mining tool to obtain valuable information from eWOM [40, 41]. This tool calculates the degree to which different categories of words are used and is based on scientific research connecting terms to personality and psychological states. Ransbotham et al. [42] employed LIWC to compare mobile WOM and nonmobile WOM. They proposed that WOM content is more affective, more concrete, and less extreme when created on mobile devices.

### Customer Q&As

Previous research on Q&A systems mainly concentrated on the context of online knowledge communities [43] and healthcare communities [44, 45]. A stream of research has investigated the motivation for Q&A activities. Social interaction [43] and self-presentation [46] have been proposed as the main intrinsic incentives for people to participate in Q&A activity, while monetary rewards are the primary extrinsic incentives [47]. Another stream of research in this area has examined the structure of Q&A systems, particularly mechanism design that matches the types of questions being asked on the platform. Identifying the characteristics of high-quality answers is crucial for the Q&A platform to improve user satisfaction [48]. Previous researchers have demonstrated multiple judgment criteria for high-quality answers, including cognitive, affective, social, utility, and information sources [49]. Lee et al. [50] extracted linguistic features through LIWC dictionaries. They showed that answers written more politely are more likely to be perceived as high-quality answers by the question asker due to the low face threat.

Customer Q&As are still an emerging topic in e-commerce research that has rarely been empirically explored. Khern-am-nuai et al. [51] investigated the economic implications of customer Q&As. The authors found that questions hurt product sales, while answers, particularly the depth of the answers, positively impact sales. In addition, the fraction of questions with at least one answer has a positive and significant impact on product sales. Banerjee et al. [7] proposed the first empirical research that considered the interaction between customer Q&As and online reviews. They found that customer Q&As could complement online reviews: Customer Q&As mitigate product fit uncertainty, leading to better matches between consumers and consumers, which leads to a rise in product ratings. Specifically, for products suffering from fit mismatch, receiving Q&As can improve their subsequent ratings by approximately 0.1 to 0.5, and the fraction of negative reviews that discuss fit-related issues also declines.

## Hypotheses development

### Online reviews and product sales

In online shopping, consumers face strong perceived uncertainty before making purchase decisions and seek for online reviews to obtain useful information about the quality or usage of the product [2, 52]. Most e-commerce platforms invite consumers to write online reviews after purchasing products, and even if consumers do not upload their reviews in time, the platforms will default to positive reviews posted by consumers. Therefore, the number of online reviews can reflect the popularity of the product. The higher the number of online reviews, the more likely consumers are to buy the product due to the information cascade effect [14]. Online review valence reflects the sentiment and attitude of consumers who have already purchased the product [34]. The more positive the review valence, the more it can evoke emotions and purchase intentions of potential consumers, which can lead to higher product sales. Thus, we hypothesize:

*H1a*: *Online review volume positively influences product sales*.
*H1b*: *Online review valence positively influences product sales*.

### Customer Q&As and product sales

Customer Q&A is an emerging form of eWOM that can be divided into two aspects: questions and answers. In the context of online shopping, asking questions is an effective way for consumers to seek information or help, which can help reduce their potential consumer perceived uncertainty, solve the information asymmetry issue, and promote product sales [51]. However, from another perspective, questions may also act as a negative information signal that makes consumers question the quality of the product, causing them to postpone or abandon their purchase decision [53]. Therefore, the answer rate of questions in e-commerce platforms is crucial: the questions asked by consumers will only generate relevant information value if they are answered. Therefore, we propose the following hypothesis:

*H2a*: *Question volume positively influences product sales*.
*H2b*: *Response rates of questions positively influence product sales*.

In e-commerce platforms, the answers in customer Q&As play a similar role to online reviews, representing the opinions of consumers who have already purchased the product, which provides more useful information about the product and helps potential consumers’ purchase decisions [51]. However, it should be noted that there are some differences in the content of customer Q&As and online reviews, as customer Q&As are more related to product specific attributes and characteristics, while online reviews are more related to consumers’ product usage experience, so whether there are differences in the influence of these two types of eWOM on product sales needs to be further explored. Thus, we hypothesize the following:

*H2c*: *Answer volume positively influences product sales*.
*H2d*: *Answer valence positively influences product sales*.

### Online reviews and customer Q&As

It has been found that online reviews and customer Q&As play different roles in the consumer decision making process in e-commerce platforms: online reviews can address consumers’ uncertainty about product quality, while customer Q&As can address inconsistencies in product matching [7]. Consumers who have already purchased a product may face various problems when using it, and customer Q&A can serve as a kind of after-sales service that helps consumers solve their problems. Therefore, customer Q&A can contribute to consumer satisfaction and make the valence of online reviews more positive. In addition, it has been shown that consumers will refer to other consumers’ eWOM content before posting eWOM [54]. The greater number of online reviews or customer Q&As in e-commerce platforms can enhance consumers’ social presence and increase the depth and breadth of their information sharing. We conjecture that there may be a mutually promoting effect between the two types of eWOM (online reviews and customer Q&As). Building on this, we hypothesize the following:

*H3*: *There is a mutually reinforcing relationship between online reviews and customer Q&As*.

## Data

### Data collection

The data for this study were collected from Amazon.com, primarily because Amazon is the world’s largest online retail platform, which offers an online review system and a customer Q&A system. To obtain the datasets for analysis in our study, we complied with Amazon’s API restrictions and did not violate any of the terms of the Amazon Developer Agreement and Policies. For the product types, we selected high-involvement products as the research object. Considering the number of choices available, the product complexity, and the high price, consumers are more likely to conduct extensive information searches for high-involvement products [55]. When purchasing low-involvement products, consumers may rely on a single type of eWOM information to make a purchase decision. Therefore, we selected laptops as the research object, a typical example of a high-involvement product. We collected daily data on laptop sales on Amazon.com from August 2019 to November 2019. The data included the best-seller list, online review data (text and timestamp), customer Q&A data (text and timestamp), release dates, and prices. To ensure data sufficiency of this study, we excluded products that had been on the best-seller list for fewer than three days. We obtained a total of 105 products with 7205 online reviews, 6365 customer Q&A questions, and 7419 answers.

## Variables and description statistics

### Online review variables

To explore the interaction relationship among online reviews, customer Q&As, and product sales, we need to construct variables at the aggregate level. We aggregated the online review data to the product week level. Review volume was measured as the cumulative number of consumer reviews in a certain period. For the measurement of review valence, previous studies mainly used consumer ratings [56]. However, Amazon does not provide a rating function in customer Q&As. As the measures of online review valence and customer Q&A valence in this study should be consistent, ratings cannot be the measurement of eWOM valence.

Therefore, we referred to Hu et al. [57] and coded eWOM valence as the absolute value of the difference between positive affective words and negative affective words divided by the total number of affective words. To extract the affective linguistic features from eWOM content, we processed the full text of online reviews and Q&As using the LIWC program [58]. LIWC measures the number of words in a given text that reflect particular linguistic or psychological processes and spoken language categories, which has been widely used in IS and marketing research [5, 42].

### Customer Q&A variables

We divided the customer Q&As into questions and answers. Using the same measurement as for online reviews, we constructed three variables: question volume, answer volume, and answer valence. As most questions have neutral valences, we did not include question valence.

In addition, we added the response rate, which is calculated by the proportion of questions that are answered. In other words, it represents how much uncertainty has already been resolved by the Q&A platform in response to other customers’ concerns. We argue that more unanswered questions about a product will lead to a high level of perceived uncertainty, which, in turn, discourages consumers from purchasing that product. Furthermore, because online retailers can respond to questions, response rates can be somewhat indicative of the management response, which can have an impact on consumer purchases [28, 59].

### Product sales

Due to the difficulty of obtaining actual real-time sales, we used sales rank as a proxy for actual sales. Sales rank and actual sales follow a log-linear relationship, thus the marginal effect on sales rank can be interpreted as an effect on sales [31]. On Amazon’s best-seller list, lower sales ranks indicate higher sales. To represent product sales, we used the reciprocal of sales rankings, considering the negative relationship between sales rankings and sales.

### Description statistics

Table 1 shows the descriptive statistics of the variables. In terms of eWOM generation, the number of online reviews and the number of customer questions and answers are about the same, which implies that although customer Q&As are an emerging type of eWOM, they have gained a significant following among consumers. For valence, online reviews are much greater than customer Q&A answers. This occurs mainly due to the differences in content between these two types of eWOM, with online reviews focusing more on personal experiences and customer Q&As focusing more on product issues. The average response rate for customer Q&As is 23.6%, suggesting that some questions remain unanswered.

**Table 1:** Description statistics.

Variable	Mean	SD	Min	Max
Sales	0.056	0.118	0.010	1
Review volume	6.269	6.258	1	53
Review valence	0.542	0.271	0.000	1
Question volume	5.873	6.395	1	61
Answer volume	5.846	6.259	1	62
Answer valence	0.239	0.274	0.000	1
Response rate	0.236	0.321	0.000	1

## Methodology

### PVAR model

Next, we examined the dynamic relationship among online reviews, customer Q&As, and sales. We used PVAR as our econometric model, which permits us to account for the interrelationships among these periods to quantify the effect of one variable on another. The PVAR model is an extension of the vector autoregression (VAR) model; it combines the VAR model with panel data, allows estimations for multiple cross-sections of data, and considers unobserved individual heterogeneity [60].

We choose the PVAR model mainly for the following reasons. First, the endogeneity problem is one of the methodological challenges in this study. For instance, eWOM volume can both be a cause and an outcome of product sales. Besides reverse causality issues, omitting variables can also cause endogeneity. For example, consumer product attribute preferences and other individual characteristics that are not easily captured may be associated with eWOM valence, which can affect product sales. PVAR can handle the endogeneity issue by including the regression’s lagged variables, such as sales from a previous time in the instrument, without imposing unnecessary restrictions. PVAR also allows the inclusion of a product’s fixed effects to overcome unobserved individual heterogeneity. Second, the impulse response function (IRF) can capture the delayed impacts of online reviews and customer Q&As on product sales and estimate the overall reaction of one endogenous variable to a shock on another variable during a certain period.

### Model specification

Based on the previous literature, we conducted the PVAR model as follows:

[SalestRevvoltRevvaltQuevoltAnsvoltAnsvaltRatet]=∑Jj=1[β11t−jβ12t−jβ13t−jβ14t−jβ15t−jβ16t−jβ21t−jβ22t−jβ23t−jβ24t−jβ25t−jβ26t−jβ31t−jβ32t−jβ33t−jβ34t−jβ35t−jβ36t−jβ41t−jβ42t−jβ43t−jβ44t−jβ45t−jβ46t−jβ51t−jβ52t−jβ53t−jβ54t−jβ55t−jβ56t−jβ61t−jβ62t−jβ63t−jβ64t−jβ65t−jβ66t−jβ71t−jβ72t−jβ73t−jβ74t−jβ75t−jβ76t−j][Salest−jRevvolt−jRevvalt−jQuesvolt−jAnsvolt−jAnsvalt−jRatet−j]+Control+[εSales,tεRevvol,tεRevval,tεQuesvol,tεAnsvol,tεAnsval,tεRate,t]

where Sales represents product sales, Revvol represents the review volume, Revval represents the review valence, Quesvol represents the question volume, Ansvol represents the answer volume, Ansvol represents the answer valence, and Rate represents the response rate. Let i denote products, and t denote the time. j denotes the optimal lag length. Control represents the control variable of this study, including product price and product release time. The variable ε is the idiosyncratic error term with a mean of zero. To finalize the model, we need to identify the optimal number of lags (j). We used various information criteria, such as the Akaike information criterion (AIC), the Bayesian information criterion (BIC), and the Hannan–Quinn information criterion (HQIC), and determined the optimal lag is one.

### Stationarity test

Before performing the PVAR estimation, we need to verify the stationarity of the endogenous variables in our data set [61]. Because our data are unbalanced, certain panel unit-root tests could not be utilized, because they require strongly balanced data. Referring to Lin et al. [62], we used the ADF-Fisher test, which is particularly suitable for unbalanced panel data. The results of the panel unit root tests are shown in Table 2. For all seven variables, the null hypothesis of having a unit root is rejected, which indicates that all the variables were stationary.

**Table 2:** Results of panel unit root tests.

Variables	ADF-fisher	Conclusion
Sales	419.241***	Stationary
Revvol	431.042***	Stationary
Revval	499.101***	Stationary
Quesvol	434.223***	Stationary
Ansvol	476.665***	Stationary
Ansval	567.080***	Stationary
Rate	509.011***	Stationary

* p < 0.1

** p < 0.05

*** p < 0.01

## Estimation results

### PVAR analysis

Using the Abrigo and Love [61] code for STATA, we applied the PVAR model and obtained the coefficient estimates displayed in Table 3. We report the results of the impact of online reviews and customer Q&As on product sales in Table 3, Column 1, which shows that the two types of eWOM have different influence mechanisms on product sales. For online reviews, volume positively influences sales (0.082, p<0.05), while for customer Q&As, answer valence positively influences sales (0.008, p<0.05), thus supporting H1a and H2d. However, the effects of review valence, question volume, response rate, and answer volume on product sales are not significant, thus H1b, H2a, H2b, and H2c are not supported. The positive volume effect of online reviews can be attributed to the information cascade effect: A large number of online reviews signals to potential consumers that a product is popular, making them more likely to follow others’ purchasing decisions. We speculated that the volume effect of customer Q&As is not significant perhaps because of the platform’s display format. The Amazon and Taobao.com platforms show only one answer, and consumers need to click the button “see more answers” if they want to read all the answers. the positive valence effect of eWOM on product sales was reflected in customer Q&As rather than in online reviews. This could be because online reviews, many of which may be published by the network water army, are suspected of being manipulated [63]. The questions in customer Q&As are mainly randomly pushed by the platform to consumers who have bought the product. Therefore, for potential consumers, the content of customer Q&As may be more credible than online reviews.

**Table 3:** Estimation results from the PVAR model.

Variables	(1)	(2)	(3)	(4)	(5)	(6)	(7)
	Sales_t_	Revvol_t_	Revval_t_	Quesvol_t_	Ansvol_t_	Ansval_t_	Rate_t_
Sales_t-1_	0.728***	0.597**	0.042	0.275	0.361	-1.252	0.556
	(0.090)	(0.261)	(0.502)	(0.262)	(0.301)	(1.309)	(0.731)
Revvol _t-1_	0.0827**	0.150	-0.061	0.025	0.036	0.489	-0.052
	(0.038)	(0.112)	(0.285)	(0.113)	(0.126)	(0.545)	(0.312)
Revval _t-1_	0.0145*	-0.001	-0.021	0.022	-0.010	0.146	-0.023
	(0.008)	(0.027)	(0.053)	(0.020)	(0.028)	(0.107)	(0.070)
Quesvol _t-1_	-0.005	-0.030	-0.3417**	0.261***	0.2007**	0.117	0.179
	(0.025)	(0.075)	(0.167)	(0.071)	(0.084)	(0.371)	(0.202)
Ansvol _t-1_	-0.009	0.2047**	0.4837**	0.2087**	0.289***	-0.778*	0.032
	(0.027)	(0.088)	(0.218)	(0.090)	(0.105)	(0.428)	(0.254)
Ansval _t-1_	0.0087**	0.001	0.029	0.003	0.016	0.237***	-0.018
	(0.004)	(0.012)	(0.032)	(0.012)	(0.014)	(0.062)	(0.035)
Rate _t-1_	0.004	-0.037*	-0.013	-0.010	-0.029	-0.015	0.034
	(0.006)	(0.020)	(0.052)	(0.021)	(0.024)	(0.107)	(0.059)
Control	Yes	Yes	Yes	Yes	Yes	Yes	Yes
Observations	763	763	763	763	763	763	763

* p < 0.1

** p < 0.05

*** p < 0.01

The impact of customer Q&As and other factors on online reviews is shown in Column 2 and Column 3 in Table 3. The results show that the answer volume has a significant and positive impact on the volume of online reviews (0.204, p<0.05). We argue that a large number of answers can enhance an online review publisher’s social presence, which leads to a stronger intention to share online reviews. In addition, we found that the answer volume can positively influence the valence of online reviews (0.483, p<0.05). More answers can solve the difficulties consumers encounter in using the product, resulting in a higher valence of online reviews. We also found that question volume can negatively influence the valence of online reviews which also verified our conjecture that more questions may raise consumer concerns about product quality issues. The impact of online reviews and other factors on customer Q&As is shown in Column 4 through Column 7 in Table 3. The volume and valence of online reviews have no significant effect on all the Q&A metrics. Therefore, based on the above results, H3 was partially supported.

In addition to the results above, we obtained other conclusions. We found that although the response rate does not have an impact on product sales, online reviews and customer Q&As. Then, we verified the positive impact of product sales on the volume of online reviews (0.597, p<0.05), since more people buying the product also means more people can have the opportunity to post online reviews. Finally, the results also indicate the autoregressive effect of sales (0.728, p<0.05), question volume (0.261, p<0.05), answer volume (0.289, p<0.05), and answer valence (0.237, p<0.05), which means past values significantly influence present values.

### Impulse response functions

**Fig 2:**
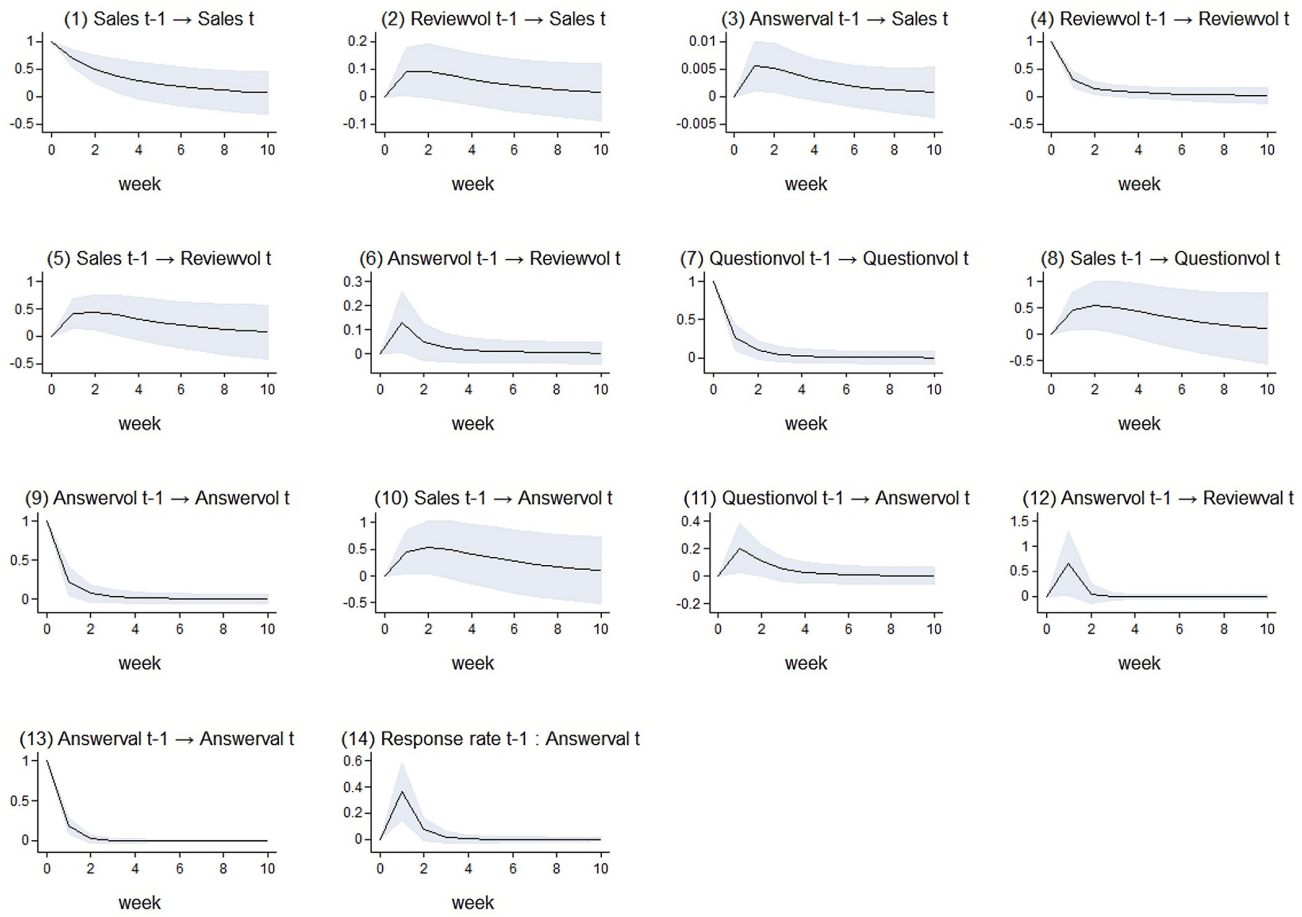
Impulse response functions (impulse→response).

We complement the regression estimates with an analysis of the corresponding IRFs. The IRFs allow us to investigate the response of one variable to a shock in another variable and to examine whether the impact is temporary or longer term. Fig 2 presents the 12 possible IRFs for the significant effects estimated in the PVAR model. Each plot in Fig 2 can be interpreted as depicting the corresponding response of a dependent variable over time to a one-standard-deviation shock in another dependent variable in the previous period, while all other variables remain constant.

Fig 2(2) and 2(3) show the dynamic impact of review volume and answer valence on product sales, respectively. Both increased gradually from the first week. Regarding influence intensity, the impact of review volume is stronger than that of answer valence. Fig 2(5), 2(7), and 2(9) visualize the dynamic impact of answer volume on review volume, review valence, and question volume, respectively. Both peaks in the first week and these effects have stabilized since the second week.

The impact of product sales on review volume is shown in Fig 2(4). This effect gradually increases from the first week. Fig 2(6) shows the negative impact of question volume on review valence. It increases in the first week and then gradually declines towards 0. Fig 2(10) presents the impact of question volume on answer volume. This effect also peaks in the first week and then remains in a relatively stable trend since the second week.

Fig 2(1) shows the autoregression of sales; it gradually and slowly increases from the first week. Fig 2(8), 2(11), and 2(12) show the autoregression of question volume, answer volume, and answer valence, respectively. Different from the autoregression of sales, the autoregression of review volume peaks in the first week, and attenuates sharply from week 2.

## Discussion

### Conclusion

Online reviews and customer Q&As are two typical types of eWOM widely adopted by retailers and embraced by consumers. However, existing studies primarily focus on online reviews and rarely include other forms of eWOM. Based on secondary data from Amazon, we conducted a PVAR model to explore the interaction among online reviews, customer Q&As, and product sales. Results indicate that online reviews and customer Q&As have two different influencing mechanisms on product sales. In particular, review volume and answer valence positively influence sales. There are conflicting findings in existing eWOM research: some studies suggest that eWOM volume positively impacts product sales [32, 64], while others propose that eWOM valence positively impacts product sales [33, 65]. Our result provides a possible explanation for the eWOM research paradox. Based on this, we further explored how the impacts of online reviews and customer Q&As on product sales changed over time. Interestingly, the dynamics of these two impacts appear to be similar; both peaked in the first week and then gradually declined to zero. In terms of intensity, online reviews have a stronger impact on product sales than customer Q&As.

Moreover, we found a facilitating effect of Q&A answers on online reviews. On one hand, the volume of Q&A answers can significantly increase the valence of online reviews. This finding is consistent with Banerjee et al.’s [7] that customer Q&As complement online reviews. Customer Q&As can solve consumers’ problems matching product features and increase the emotional tendency of subsequent consumers. On one hand, we further found that the answer volume had a significant positive impact on the online review volume. It has been found that there is a social learning effect in the eWOM generation [25, 54], and our finding extends the existing literature by proposing that the social learning effect can scan different eWOM types. On the temporal dimension, the influence of customer Q&As on online reviews peaked during week 1, diminished rapidly from week 1 to week 2, and gradually declined after week 2.

### Theoretical contributions

This study contributes to theory and research in several ways. First, the performance impact is a crucial issue in eWOM research. Many scholars have focused on this issue, but there is still a lack of consistent conclusions. The present results go beyond previous reports, showing that online reviews and customer Q&As have different influence mechanisms on product sales. Online reviews have a volume effect on sales, whereas customer Q&As have a valence effect on sales. This conclusion highlights the importance of multi-type eWOM research and provides a possible explanation for the paradox of eWOM research. Second, we further explored the interaction between online reviews and customer Q&As. The results show that the relationship between online reviews and customer Q&As is complementary rather than competitive. Specifically, answer volume positively impacts the volume and valence of online reviews. This finding enriches the theoretical understanding of media richness and makes a considerable theoretical contribution to the literature on the interaction between different types of eWOM. Third, we examined the role of customer Q&As in an e-commerce environment from multiple perspectives, including the question volume, the answer volume, the answer valence, and the response rate. This study addresses the gaps in current research related to customer Q&As and lays the groundwork for further research.

### Practical implications

The findings of this study also have significant implications for practice. First, online platforms or retailers should have a different focus when managing online reviews versus customer Q&As. For online reviews, retailers can incentivize consumers to generate online reviews through monetary or credit rewards, considering that the volume of online reviews is still a major driving factor for product sales. For customer Q&As, retailers need to monitor answers in real time, promptly clarify and explain negative answers, and report malicious answers to avoid a negative impact on sales. Second, customer Q&As can help eliminate the uncertainty of product matching faced by consumers and further enhance their post-purchase evaluations. Online platforms or retailers should be aware of this and can take the initiative to ask questions about possible difficulties or problems in using their products, as well as provide detailed explanations for these questions. In addition, the response rate will also have an impact on future respondents. Therefore, it is vital for retailers to step in and promptly answer unanswered questions, as this somehow reflects the after-sales services of retailers and will directly affect respondents’ emotions.

### Limitations and future research

This work is subject to some inevitable limitations, which provide promising directions for future research. First, due to the unavailability of real-time sales data from online retailers, we used only the Amazon best-seller ranking as a proxy variable for product sales available to the public. Although previous studies have established that sales rank tends to be a reasonable measure of product sales, real-time data can provide flawless research conclusions and management insights. Second, for the product types, this research was limited to high-involvement products. Future research can test the robustness of the present findings for products with low involvement or examine the moderating role of product types in the eWOM effect on sales. Third, consumers may have different information-processing paths in information adoption and have different preferences for information sources. However, this issue was not considered in this study. One potential extension would be to study how consumers choose when faced with online reviews and customer Q&As, and how they process information from multi-type eWOM.

## Supporting information

S1 Data (CSV)

S1 File (DOCX)
