# Supplementary material for: Online reviews, customer Q&As, and product sales: A PVAR approach
Source: PLoS One. 2023 Nov 17;18(11):e0290674. doi: 10.1371/journal.pone.0290674 (PMC10655965; doi:10.1371/journal.pone.0290674)
Supplement: S1 File — (DOCX) [file pone.0290674.s002.docx]

***log-transformation***

gen lnsales=log(sales)

gen lnrevvol=log(revvol)

gen lnrevval=log(revval)

gen lnquesvol=log(quesvol)

gen lnansvol=log(ansvol)

gen lnansval=log(ansval)

gen lnrate=log(rate)

gen lndays=log(days)

gen lnprice=log(price)

***Table 2: Unit Root Tests***

xtset id week

xtunitroot fisher lnsales,dfuller lags(1) drift

xtunitroot fisher lnrevvol,dfuller lags(1) drift

xtunitroot fisher lnrevval,dfuller lags(1) drift

xtunitroot fisher lnquesvol,dfuller lags(1) drift

xtunitroot fisher lnansvol,dfuller lags(1) drift

xtunitroot fisher lnansval,dfuller lags(1) drift

xtunitroot fisher lnrate,dfuller lags(1) drift

***Table 3: PVAR Regression***

pvar lnsales lnrevvol lnrevval lnquesvol lnansvol lnansval lnrate, exo(lndays lnprice)

***Figure 2: Impulse Response Functions***

pvarirf, oirf cumulative iteration(100)
